# TeachOpenCADD 2022: open source and FAIR Python pipelines to assist in structural bioinformatics and cheminformatics research

**DOI:** 10.1093/nar/gkac267

**Published:** 2022-05-07

**Authors:** Dominique Sydow, Jaime Rodríguez-Guerra, Talia B Kimber, David Schaller, Corey J Taylor, Yonghui Chen, Mareike Leja, Sakshi Misra, Michele Wichmann, Armin Ariamajd, Andrea Volkamer

**Affiliations:** In Silico Toxicology and Structural Bioinformatics, Institute of Physiology, Charité – Universitätsmedizin Berlin, corporate member of Freie Universität Berlin and Humboldt-Universität zu Berlin, Germany; In Silico Toxicology and Structural Bioinformatics, Institute of Physiology, Charité – Universitätsmedizin Berlin, corporate member of Freie Universität Berlin and Humboldt-Universität zu Berlin, Germany; In Silico Toxicology and Structural Bioinformatics, Institute of Physiology, Charité – Universitätsmedizin Berlin, corporate member of Freie Universität Berlin and Humboldt-Universität zu Berlin, Germany; In Silico Toxicology and Structural Bioinformatics, Institute of Physiology, Charité – Universitätsmedizin Berlin, corporate member of Freie Universität Berlin and Humboldt-Universität zu Berlin, Germany; In Silico Toxicology and Structural Bioinformatics, Institute of Physiology, Charité – Universitätsmedizin Berlin, corporate member of Freie Universität Berlin and Humboldt-Universität zu Berlin, Germany; In Silico Toxicology and Structural Bioinformatics, Institute of Physiology, Charité – Universitätsmedizin Berlin, corporate member of Freie Universität Berlin and Humboldt-Universität zu Berlin, Germany; In Silico Toxicology and Structural Bioinformatics, Institute of Physiology, Charité – Universitätsmedizin Berlin, corporate member of Freie Universität Berlin and Humboldt-Universität zu Berlin, Germany; In Silico Toxicology and Structural Bioinformatics, Institute of Physiology, Charité – Universitätsmedizin Berlin, corporate member of Freie Universität Berlin and Humboldt-Universität zu Berlin, Germany; In Silico Toxicology and Structural Bioinformatics, Institute of Physiology, Charité – Universitätsmedizin Berlin, corporate member of Freie Universität Berlin and Humboldt-Universität zu Berlin, Germany; In Silico Toxicology and Structural Bioinformatics, Institute of Physiology, Charité – Universitätsmedizin Berlin, corporate member of Freie Universität Berlin and Humboldt-Universität zu Berlin, Germany; In Silico Toxicology and Structural Bioinformatics, Institute of Physiology, Charité – Universitätsmedizin Berlin, corporate member of Freie Universität Berlin and Humboldt-Universität zu Berlin, Germany

## Abstract

Computational pipelines have become a crucial part of modern drug discovery campaigns. Setting up and maintaining such pipelines, however, can be challenging and time-consuming—especially for novice scientists in this domain. TeachOpenCADD is a platform that aims to teach domain-specific skills and to provide pipeline templates as starting points for research projects. We offer Python-based solutions for common tasks in cheminformatics and structural bioinformatics in the form of Jupyter notebooks, based on open source resources only. Including the 12 newly released additions, TeachOpenCADD now contains 22 notebooks that cover both theoretical background as well as hands-on programming. To promote reproducible and reusable research, we apply software best practices to our notebooks such as testing with automated continuous integration and adhering to the idiomatic Python style. The new TeachOpenCADD website is available at https://projects.volkamerlab.org/teachopencadd and all code is deposited on GitHub.

## Graphical Abstract

**Graphical Abstract:**
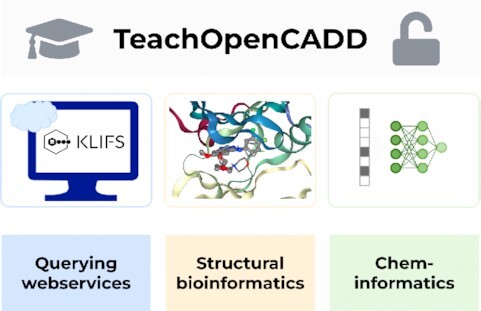
TeachOpenCADD provides open source and FAIR Python pipelines to assist in structural bioinformatics and cheminformatics research.

## INTRODUCTION

Computational methods play an integral role in the design-make-test-analyze (DMTA) cycle that drives real-world drug design projects (1). To address questions raised during this cycle, a single method does not suffice to deliver an answer; instead, a pipeline combining different methods can produce complementary and useful insights. Setting up such complex pipelines, however, can be difficult and time-consuming for many reasons: the scientist may not have had the training necessary to tackle these tasks (2), tools and their usage are constantly evolving (or becoming deprecated), and feeding the output from one tool into another is often not straightforward. On top of these considerations, sustainable pipelines need to be findable, accessible, interoperable, and reusable (FAIR principles (3))—not only today but in many years from now—to drive reproducible research.

In 2019, we launched the teaching platform TeachOpenCADD (4) on GitHub to help face these challenges. TeachOpenCADD teaches by example how to build Python pipelines with open source resources used in the fields of cheminformatics and structural bioinformatics to answer central questions in computer-aided drug design (CADD). With these ready-to-use pipelines, we target students and teachers who need training material for CADD-related topics, as well as researchers who need a template or an inspiration to tackle their research questions. The theoretical and practical aspects of each topic are covered in an interactive Jupyter notebook (5). This setup makes it easy for users from different fields to understand the computational concepts and to get started with hands-on Python programming. We call these Jupyter notebooks *talktorials* (talk + tutorial) because their format is suited for presentations as well. The initial stack of talktorials T001–T010 covers common CADD tasks involving webserver queries, cheminformatics, and structural bioinformatics (4). We show how to fetch chemical and structural data from the ChEMBL (6) and PDB (7,8) databases and how to encode, filter, cluster, and screen such datasets to find novel drug candidates and off-targets (4). The talktorials are inspired by several online resources recommended for further reading such as Teach-Discover-Treat and CDK (9,10) and the blogs Practical Cheminformatics, RDKit blog, and Is live worth living?. Over the last two years, the TeachOpenCADD GitHub repository underwent many additions and changes: we now have more than doubled our content and extended the application of software best practices rigorously. The full collection of talktorials is easily accessible on the new TeachOpenCADD website. We comply with software best practices regarding the code style as well as maintenance and facilitate installation with a dedicated conda package.

## NEW TALKTORIALS

The new stack of talktorials showcases data acquisition from additional CADD-relevant databases, adds many examples for structure-based tasks, and extends the cheminformatics side with straightforward deep learning (DL) applications. Our example use case is the EGFR kinase (19) but the talktorials are easily adaptable to other targets as long as sufficient data is available. Besides the domain-specific resources described below, we rely in all talktorials on established Python packages for data science and visualization such as NumPy (20), pandas (21), scikit-learn (22), matplotlib (23), and seaborn (24).

### Webservices queries

Over the last decades, the scientific community has produced an incredible amount of data and analysis software, and adapted modern technologies to make these resources easily available via online webservices (25). However, it might not always be obvious to the beginner how to use a web application programming interface (API) to access such data and how to integrate them into larger pipelines. TeachOpenCADD dedicates several talktorials to the usage of different webservers relevant for the life sciences.

In the first TeachOpenCADD release from 2019, we already showed how to query the ChEMBL (6) and PDB (7,8) databases. From the ChEMBL webservice, compounds and bioactivities are fetched for the EGFR kinase using the ChEMBL webresource client (26) (T001). This dataset is used in many downstream talktorials for common cheminformatics tasks (T002-T007). From the PDB webservice, we fetch a set of EGFR kinase structures based on criteria such as ‘ligand-bound structures from X-ray experiments with a resolution <3.0 Å’ using the biotite (27) and PyPDB (28) (T008) packages.

**Figure 1.**
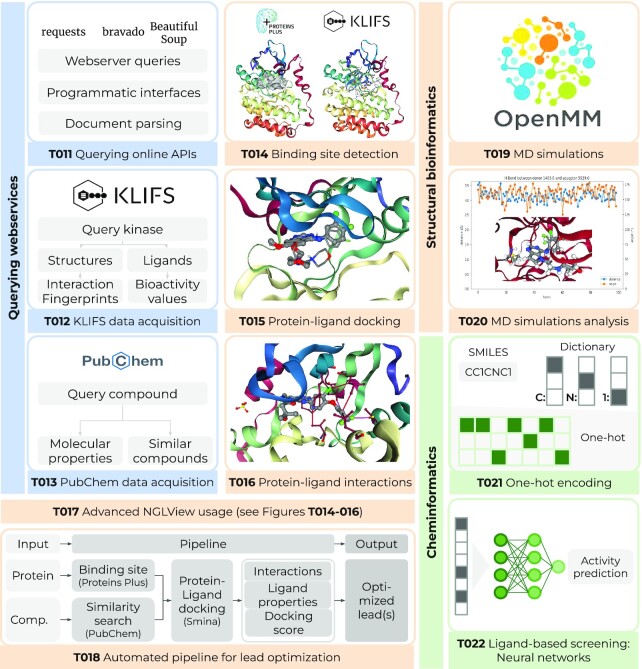
Overview of 12 new talktorials. (i) Querying webservices (blue): T011 gives a broad introduction to programmatic access to webservices from Python, T012 and T013 demonstrate how to query the KLIFS (11) and PubChem (12) databases for kinase and compound data, respectively. (ii) Structural bioinformatics (orange): T014 detects the binding site in an EGFR kinase structure and compares the prediction to the binding site defined by KLIFS (11). T015 performs a re-docking for an EGFR–ligand complex with Smina (13). T016 detects protein–ligand interactions in an EGFR–ligand complex structure with PLIP (14). T017 introduces basic and advanced usages of the molecular visualization tool NGLView (15), used throughout most of TeachOpenCADD’s talktorials. T018 outlines a fully automated lead optimization pipeline: Based on an input structure, the pocket is detected and a set of compounds similar to a selected ligand are fetched from PubChem (12). These compounds are docked into the selected binding site. The most promising compounds w.r.t. docking scores and interaction profiles are proposed as optimized compounds. T019 demonstrates how to set up and run a molecular dynamics (MD) simulation on Google Colab with OpenMM (16). T020 analyzes the resulting MD trajectory with a focus on the root-mean-square deviation (RMSD) between trajectory frames and the dynamics of protein-ligand interactions using MDAnalysis (17,18). (iii) Cheminformatics (green): T021 exhibits the steps to numerically encode a small molecule from its SMILES representation. T022 lays the groundwork for deep learning and focuses on a simple feed-forward neural network for activity prediction using molecular fingerprints.

In the latest release, we now have added three more notebooks covering the usage of additional online API webservices (Figure 1, T011–T013).

#### T011: Querying online API webservices

We added a broad introduction on how to programmatically use online webservices from Python with a focus on REST services and web scraping. The usage of several libraries is demonstrated; e.g. we use requests to retrieve content from UniProt (29), bravado to generate a Python client for OpenAPI-compatible services — exemplified for the KLIFS database (11)—, and Beautiful Soup to scrape (parse) HTML content from the web.

#### T012: Data acquisition from KLIFS

KLIFS (11) is a kinase database gathering information on experimental kinase structures and interacting inhibitors. The talktorial shows how to quickly fetch data from KLIFS given a query kinase or ligand. For example, we spot frequent key ligand-interactions in EGFR based on KLIFS interaction fingerprints and we assess kinome-wide bioactivity values for the inhibitor gefitinib. These queries are demonstrated by using the KLIFS OpenAPI directly with bravado, or by using the KLIFS-dedicated wrapper OpenCADD-KLIFS (30), implemented in the Python package OpenCADD.

#### T013: Data acquisition from PubChem

PubChem (12) is a database holding chemical information on over 100 million compounds. We demonstrate how to fetch data from PubChem’s PUG-REST API (31), given the name or SMILES (32) of a query ligand. For example, we show how to fetch molecular properties for a ligand of interest by name (aspirin) and how to query PubChem for the most similar compounds given a query SMILES (gefitinib).

#### Data acquisition case study

**Figure 2.**
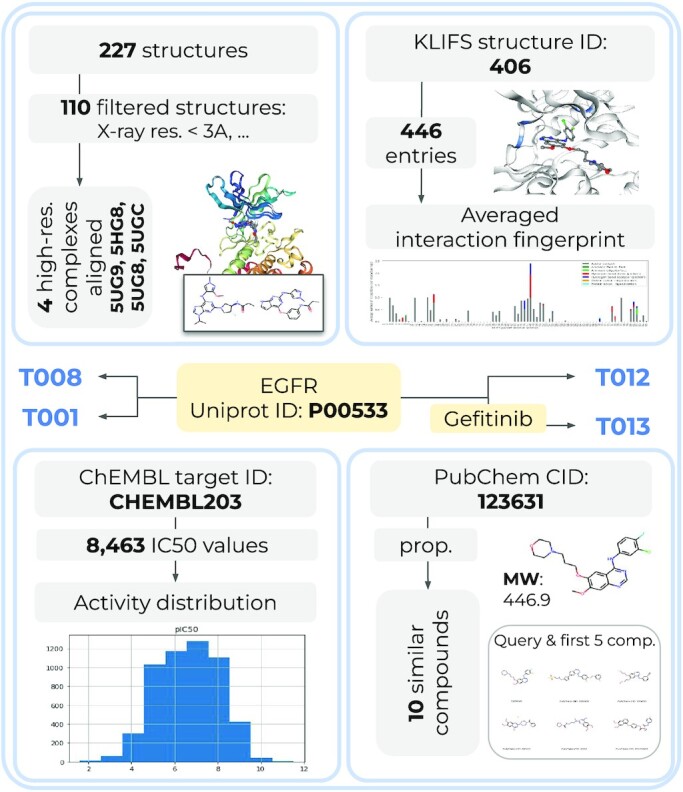
Data and information that can be automatically gathered for the EGFR kinase using the different web query talktorials as of September 2021, created based on ChEMBL v.27 (6) (T001), PDB (8) (T008), PubChem (12) (T013), and KLIFS (11) (T012). Input: yellow boxes, output: gray boxes, plots and molecule visualizations (using NGLView (15) and RDKit).

A summary of the information that can be acquired automatically for a target of interest using these webservices is exemplified in Figure 2. Using the Uniprot ID of EGFR kinase as input query only, (i) 227 available EGFR structures from the PDB can be obtained and further filtered (T008); (ii) 446 available complex structures and their interaction fingerprints can be fetched from KLIFS (T012), or (iii) a total of 8463 IC50 values of molecules measured against EGFR can be acquired from ChEMBL (T001). Finally, (iv) a PubChem query with the molecule name ‘gefitinib’ showcases how to gather ligand properties or to perform a similarity search (T013).

### Pocket detection, ligand–protein docking and interactions

During a drug discovery campaign, frequent questions are: What should I test next? Can you suggest a diverse set of small molecules likely to bind to this protein? How should I modify the lead compound to increase the binding affinity? Answering these questions involves multiple scientific observations, and thus, multiple computational steps as addressed in talktorials T014–T017. Finally, an automated pipeline is compiled (T018) to process a protein structure and a lead compound, and propose several similar ligands with optimized estimated affinities and interactions based on the docked protein-ligand structures.

#### T014: Binding site detection

First, we need to know where ligands may bind to a protein of interest. Sometimes the binding site is known from experimental protein-ligand structures. If only experimental apo structures are available, putative binding sites can be predicted with computational methods. We demonstrate how to use the REST API of the ProteinsPlus webserver (33) to detect the main pocket of an EGFR structure using the DoGSiteScorer (34) pocket detection algorithm. To validate our results, the predicted pocket is compared with the KLIFS-defined kinase pocket, which encompasses 85 residues shown to be in contact with ligands based on X-ray complex structures (35).

#### T015: Protein–ligand docking

Next, we introduce molecular docking to predict the binding mode of a ligand to its protein target by explaining several sampling algorithms and scoring functions, as well as commenting on limitations and interpretation of docking results. The theoretical background is then applied in a re-docking experiment aiming to reproduce the binding mode observed in a published X-ray structure of EGFR. Protein and ligand are prepared using Pybel (36), the ligand is docked into the protein using Smina (13), and finally, the docking poses are visually inspected using NGLView (15). We refer to JupyterDock for further reading on different docking protocols run from Jupyter notebooks.

#### T016: Protein–ligand interactions

Understanding which forces and interactions drive molecular recognition is important for drug design (37). In this talktorial, we give an introduction to relevant protein-ligand interactions and their programmatic detection using the protein-ligand interaction profiler PLIP (14). To this end, all interactions in an EGFR–ligand complex fetched from the PDB are detected and visualized in 3D using NGLView.

#### T017: Advanced NGLView usage

Since the molecular visualization package NGLView is invoked in many talktorials, we give a dedicated overview of its usage and show some advanced cases on how to customize residue coloring, and how to create interactive interfaces with IPyWidgets. In addition, access to the JavaScript layer NGL (38,39) is showcased to perform operations that are not exposed to the Python wrapper NGLView.

#### T018: Automated pipeline for lead optimization

All previous talktorials are composed of stand-alone tasks that can be completed independently. Proposing ligand modifications that will improve interaction patterns with target proteins in a complete end-to-end process, however, necessitates orchestration of code and concepts implemented in the previously discussed talktorials T014–T017. A docking pipeline is constructed in T018 that is comprised of both a step-by-step demonstration and a fully automated procedure. Given a query protein and a lead compound, similar ligands fetched from PubChem are suggested, which show optimized affinity estimates and interaction profiles based on generated docking poses.

#### Lead optimization case study

**Figure 3.**
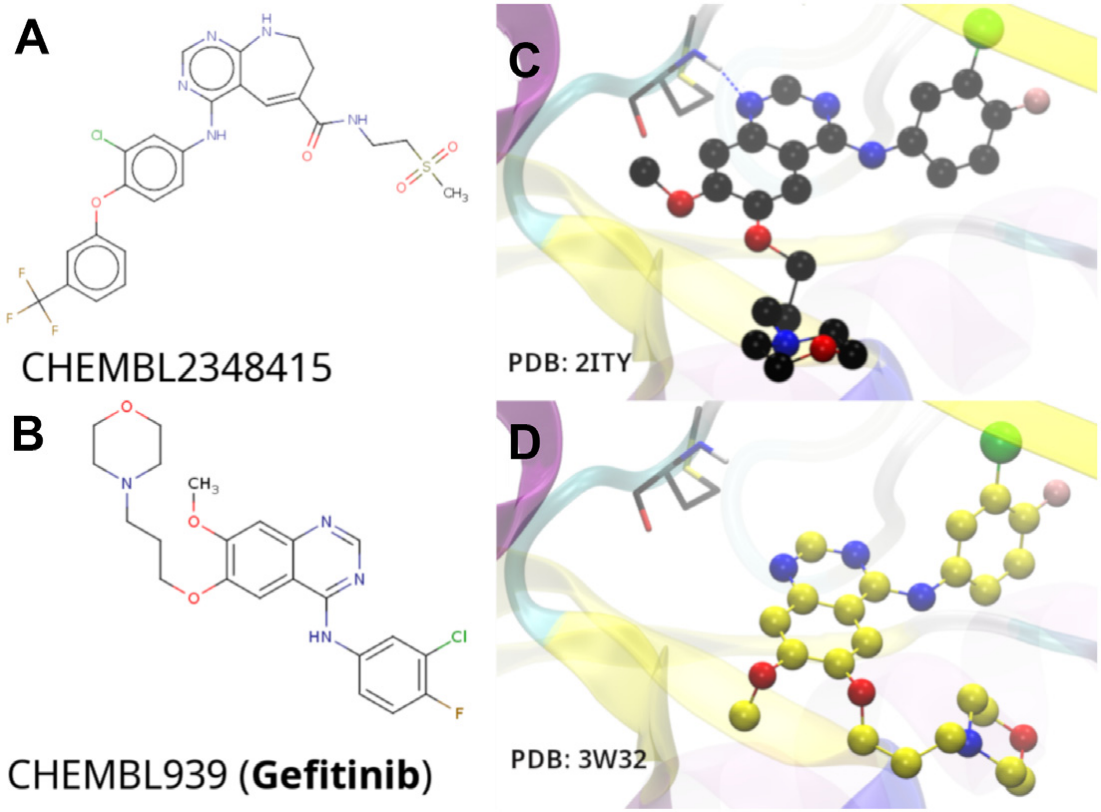
Case study for talktorial T018 depicting (**A**) 2D structure of the input ligand for the pipeline that was used with an EGFR crystal structure (PDB: 3W32, IC50 = 75nM); (**B**) 2D structure of gefitinib (IC50 = 0.17nM), an EGFR ligand found during similarity searches; (**C**) crystal structure of gefitinib co-crystallized with EGFR (PDB: 2ITY, black CPK representation); (**D**) docked pose of gefitinib (yellow CPK representation). Some segments of the protein structure have been removed for clarity. The ligand RMSD between (C) and (D) and the discovery of a higher-affinity ligand demonstrate the utility of the fully automated pipeline for early stage drug discovery.

As a case study, an EGFR crystal structure (PDB: 3W32) and its co-crystallized ligand were used as inputs for the pipeline. A similarity search led to the generation of a small library of compounds from PubChem for docking and further analysis to find compounds ideally more affine than the co-crystallized ligand. Using the pipeline, an approved breast cancer drug, gefitinib, was found in the top 50 of docked poses (Figure 3). Gefitinib (IC50 = 0.17 nM (40)) is at least an order of magnitude more affine for EGFR than the measured affinity of the input ligand (IC50 = 75 nM (41)). Gefitinib's predicted geometry was <2 Å RMSD from a crystal structure of wild-type EGFR (PDB: 2ITY). This retrospective example demonstrates the utility of a fully automated pipeline and potential application as prospective tool.

### Molecular dynamics

Experimentally resolved structures offer immense insights for drug design but can only provide a static snapshot of the full conformational space that represents the flexible nature of biological systems. Molecular dynamics (MD) simulations approximate such flexibility *in silico* with a trajectory of atom positions over a series of time steps (frames). These trajectories thereby reveal a more detailed—albeit still incomplete—picture of drug-target recognition and binding by providing access to protein-ligand interaction patterns over time (42–44). These insights can for example help in lead discovery to examine the stability and validity of a predicted ligand docking pose, and in lead optimization phases to estimate the effect of a chemical modification on binding affinity.

#### T019: MD simulations

We explain the key concepts behind MD simulations and provide the code to run a short MD simulation of EGFR in complex with a ligand on a local machine or on Google Colab with condacolab, which allows for GPU-accelerated simulations. The protein and ligand are thereby separately prepared with pdbfixer and RDKit, and subsequently combined using MDTraj (45) and openff-toolkit. The simulation is performed with OpenMM (16), a high-performance toolkit for molecular simulation. The talktorial produces a 100 ps trajectory if run on Google Colab. On a local machine, only 20 fs are generated by default to keep computational efforts reasonable. We refer to the work by Arantes *et al.* (46) for further reading on different MD protocols run with OpenMM using Jupyter notebooks on Google Colab.

#### T020: Analyzing MD simulations

We analyze and visualize the trajectory using the Python packages MDAnalysis (17,18) and NGLView. First, the protein is structurally aligned across all trajectory frames, followed by calculating the root-mean-square deviation (RMSD) for different system components, i.e. protein, backbone, and ligand. Then, we take a closer look at a selected interaction between ligand and protein atoms, showcasing the contribution of distance and angle to the hydrogen bond strengths.

### Deep learning

Machine learning and more specifically deep learning have gained in popularity over the last few decades thanks to powerful computational resources (GPUs), novel algorithms, and the growing amount of available data (47). Applications to CADD are diverse, ranging from molecular property prediction (48) to *de novo* molecular design (49). Here, the focus is the featurization of molecular entities (T021) and ligand-based screening (T022).

#### T021: One-hot encoding

In CADD, machine learning algorithms require as input a numerical representation of small molecules. Besides molecular fingerprints (see T004), a popular featurization is the SMILES notation (32). However, these representations are composed of strings and therefore cannot simply be input to an algorithm. One-hot encoding provides a solution for SMILES usage, explained in T021.

#### T022: Ligand-based screening: neural networks

We introduce the basics of neural networks and build a simple two-layer neural network. A model is trained on a subset of ChEMBL data to predict the pIC50 values of compounds against EGFR using MACCS keys as input. This talktorial is meant as groundwork for the understanding of neural networks. More complex architectures such as convolutional and recurrent neural networks will be explored in future notebooks. Such models may use the one-hot encoding of SMILES as input (50).

## BEST PRACTICES

We provide reliable and reproducible TeachOpenCADD pipelines, periodically checked via automated testing mechanisms, and a streamlined and easy-to-understand code style across all talktorials.

### Testing

Reproducibility is ensured by testing if the notebooks can run without errors and whether the output of specific operations can be reproduced. For this purpose, we use the tools pytest and nbval.

### Continuous integration

We are testing the talktorials regularly for Linux, OSX, and Windows and different Python versions on GitHub Actions. This ensures identical behavior across different operating systems and Python versions and also spots issues like conflicting dependency updates or changing outputs.

### Repository structure

The repository structure is based on the cookiecutter-cms template, which provides a Python-focused project scaffold with pre-configured settings for packaging, continuous integration, Sphinx-based documentation, and much more. We have adapted the template to our notebook-focused needs.

### Code style

We aim to adhere to the PEP8 style guide for Python code, which defines how to write idiomatic Python (Pythonic) code. Such rules are important so that new developers—or in our case talktorial users—can quickly read and understand the code. Furthermore, we use black-nb to format the Python notebooks compliant with PEP8.

## TEACHOPENCADD USAGE

There are many ways to use the talktorials. If users simply want to go through the material, they can use the read-only website version. If users would rather like to execute and modify the Jupyter notebooks, this can be done online thanks to the Binder integrations or locally using the new conda package.

### New website

Firing up Jupyter notebooks can entail unexpected complications if one wants to simply read through a talktorial. To make the access easy and fast, we launched a new TeachOpenCADD website. The website statically renders the talktorials for immediate online reading using sphinx-nb and provides detailed documentation for local usage, contributions and external resources.

### New Binder support

The Binder project offers a place to share computing environments via a single link. The environment setup of TeachOpenCADD can take a couple of minutes but does not require any kind of action on the user’s end. This access option is recommended if the user plans on executing the material but does not need to save the changes.

### New conda package

To make the local installation of TeachOpenCADD as easy as possible, we offer a conda package that ships all Jupyter notebooks with all necessary dependencies. The installation instructions are lined out in the TeachOpenCADD documentation. This access option is recommended if the user plans on adapting the material for individual use cases.

## CONCLUSION

The increasing amount of data and the focus on data-driven methods call for reproducible and reliable pipelines for computer-aided drug design (CADD). Knowing how to access and use these resources programmatically, however, requires domain-specific training and inspiration. The TeachOpenCADD platform showcases webserver-based data acquisition and common tasks in the fields of cheminformatics and structural bioinformatics. The theoretical and programmatic aspects of each topic are outlined side-by-side in Jupyter notebooks (talktorials) using open source resources only. To foster FAIR research, we apply software best practices such as testing, continuous integration, and idiomatic coding throughout the whole project. The talktorials are accessible via our website, Binder, and conda package to accommodate different use cases such as reading, executing, and modifying, respectively. We believe that TeachOpenCADD is not only a rich resource for CADD pipelines and teaching material on computational concepts and programming but as well a good example of how to set up websites, automated testing, and packaging for notebook-centric repositories. TeachOpenCADD is a living resource; problems can be voiced via GitHub issues and contributions can be made in the form of pull requests on GitHub. TeachOpenCADD is meant to grow; everyone is welcome to add new topics. Whenever you explore a new topic for your work, we invite you to fill our talktorial template with what one learns along the way and to submit it to TeachOpenCADD.

## DATA AVAILABILITY

- TeachOpenCADD website: https://projects.volkamerlab.org/teachopencadd/.
- TeachOpenCADD GitHub repository: https://github.com/volkamerlab/teachopencadd.
